# The Relation among Teeth and Maxillary Dental Arch Dimensions with Anterior Teeth Angulation and Inclination

**DOI:** 10.1155/2021/8993734

**Published:** 2021-10-05

**Authors:** Sara M. Al-Mashhadany, Jinan Eliewy Saloom, Mohammed Nahidh

**Affiliations:** Department of Orthodontics, College of Dentistry, University of Baghdad, Baghdad, Iraq

## Abstract

**Objectives:**

This study aimed at finding out whether anterior teeth angulation and inclination have a relationship with the maxillary teeth and dental arch dimensions.

**Methods:**

Fifty study models with normal occlusion were selected from the archive of the Department of Orthodontics at Baghdad Dental Faculty. Maxillary dental arch width and length at different points were determined in addition to measuring anterior teeth angulation, inclination, crown thickness, overjet, overbite, and Bolton's ratios. The unpaired *t*-test and Pearson's correlation coefficient test were used for data analysis.

**Results:**

No statistical gender differences were reported in all measurements except the dental arch widths and length where males had significantly higher mean values. Only the maxillary incisor's inclination showed a direct weak significant correlation with the total arch length.

**Conclusions:**

The inclination of upper incisors had a minimal effect on increasing dental arch length.

## 1. Introduction

One of the primary diagnostic aids utilized by orthodontists in establishing diagnosis and treatment planning is the study model [1]. Correcting a malocclusion requires achieving an optimum occlusion with good interdigitation, compatible maxillary, and mandibular teeth sizes and ideal overjet and overbite within the envelope of the basal bone [2].

Interdental stripping, expansion, extraction, and esthetic restorations can be used in correcting the malocclusions. Altering the axial inclination in mesiodistal and labiolingual direction is considered a promising method to manage the discrepancy between the tooth material and basal bone [3].

In 1972, Andrews developed the six keys for normal occlusion and clarified in the second and third keys the role of angulation and inclination in establishing optimum occlusion in addition to their effect on the dental arch length and perimeter after examining many dental models of normal occlusion individuals and subjects treated orthodontically [4]. After that, Roth [5] and others [6–10] modified the normal values recorded by Andrews, so that many prescriptions are available nowadays.

The relationship between anterior teeth angulation and inclination had been studied in different research studies. Tuverson [11] affirmed an increase in the arch length by 2 mm and 1 mm with distal angulation and lingual inclination of the upper anterior teeth, respectively. On the other hand, Hussles and Nanda [12] concluded mathematically that there was a direct relation between increased teeth angulation and height/width ratio with increasing dental arch length.

Pontes et al. [13] utilized the CBCT to study the effect of various orthodontic bracket prescriptions on the dental arch perimeter and found that the prescription with great angulation led to more space within the dental arch.

Finally, using working models, Jain et al. [14] studied the effect of upper incisors angulation and inclination on the effective dental arch perimeter and found that a 1° increase in anterior crown angulation and inclination will lead to consumption of arch perimeter by 0.012 mm and 0.021 mm, respectively.

O'Higgins et al. [15] studied the effect of maxillary incisors inclination on the dental arch length using natural and acrylic teeth. They found a direct relation between them being greater with the natural incisors which may be related to the teeth size and morphology. Moreover; a 5° increase in incisors' inclination caused approximately 1 mm increase in the dental arch length.

Up to the authors' knowledge, no study was conducted to evaluate the effect of teeth angulation and inclination on the dental arch parameters, Bolton ratio, overjet, overbite, and anterior teeth thickness on models of subjects with normal occlusion, so this study was carried out.

## 2. Methods

Approval of the scientific committee in the College of Dentistry, University of Baghdad, was taken before conducting the study.

Fifty study models of 28 females and 22 males from the students of the college and some patients who attended the same college with normal class I occlusion were retrieved from the archive of the orthodontic department. These models were trimmed, so the occlusal plane was mostly parallel to the base of the models [16].

The exclusion criteria of the cases were discrepancy of more than 0.5 mm from buccal to lingual contact, filled or missed anterior teeth, and the presence of spacing between teeth. All cases included had class I canine relationships with normal overjet and overbite, i.e., class I molar, canine, and incisor relationships [1].

The following parameters were measured on the study models using an electronic digital vernier caliper (Insize Co., USA) calibrated to the nearest 0.01 mm.

### 2.1. Bolton Ratios

The mesiodistal width of each tooth anterior to second molars was recorded at the mesial and distal contact points parallel to the occlusal surfaces. The anterior and overall Bolton's ratios were calculated as follows [17].

  Overall ratio = sum of mandibular “12” teeth/sum of maxillary “12” teeth × 100
  Anterior ratio = sum of mandibular “6” teeth/sum of maxillary “6” teeth × 100

### 2.2. The Overjet

It was measured as the horizontal distance extending from the incisal edge of the upper central incisor to the labial surface of the corresponding lower central incisor in maximum intercuspation with the digital caliper held parallel to the base [16].

### 2.3. The Overbite

It represented the vertical distance between the upper and lower central incisors in maximum intercuspation. Marks were made on the teeth of the models with a very fine pointed lead pencil. The pointed portion of the caliper was held perpendicular to the base of the models [16].

### 2.4. Crown Thickness

A crown thickness gauge was used to record the upper incisor's incisal edge thickness. There were three measurements from pencil markings 2 mm from the incisal edge per tooth: 1 mm from the mesial proximal surface, 1 mm from the distal proximal surface, and middle of the mesial-distal distance [16].

### 2.5. Anterior Teeth Angulation

It is the mesiodistal angulation of the long axis of the crown expressed in degree (plus or minus); this degree represents the angle between the long axis of the crown and a line making 90 with the occlusal plane [2] using the RaySet® device (Biaggini Medical Devices, Italy).

### 2.6. Anterior Teeth Inclination

It represented the labiolingual inclination of the long axis of the crown expressed in degree (plus or minus) corresponded to the angle between the line tangent to the middle of the labial surface of the crown and aline perpendicular to the occlusal plane [2] using the RaySet® device (Biaggini Medical Devices, Italy). For angulation and inclination, the average of the right and left anterior teeth was used in the present study.

### 2.7. Dental Arch Dimensions [18]

- Intercanine distance: the horizontal distance between cusps' tips of the upper canines
- Interpremolar distance: the horizontal distance between the buccal cusp tips of the second premolars
- Inter-first molar distance: the horizontal distance between the mesiobuccal cusp tips of the first molars
- Inter-second molar distance: the horizontal distance between the distobuccal cusp tips of the second molars
- Anterior arch length: the vertical distance from the incisal point to the intercanine distance line
- Molar vertical distance: the vertical distance from the incisal point perpendicular to a line between the mesiolingual cusp tips of the first molars
- Total arch length: the vertical distance from the incisal point to the midpoint of a line between the distobuccal cusp tips of the second molars

Data were managed with SPSS software version 25 to obtain the descriptive statistics (means and standard deviations) and inferential statistics (unpaired *t*-test and Pearson's correlation coefficient test). The probability value was set at 0.05.

## 3. Results

Table 1 provides the descriptive statistics and gender difference for all variables measured. Generally, anterior teeth inclination, angulation, Bolton's ratios, overjet, and overbite showed statistically nonsignificant gender differences. On the other hand, all measurements related to the dental arch widths and lengths were larger significantly in males.

The relations among the parameters are given in Table 2 after merging the data of both genders. Pearson's correlation coefficient test showed statistically no significant relationships among variables except the presence of a weak direct significant correlation between the inclination of upper incisors and total arch length.

## 4. Discussion

The literature described several methods to measure and study maxillary incisors' angulation and inclination and their relation with other parameters using various radiographical views and working models [11–15]. The present study focused on examining the relationship among inclination and angulation with the Bolton ratio, overjet, overbite, crown thickness, and dental arch length and width on models of subjects with normal occlusion unlike the previous studies, so investigating the effect of incisors' inclination and angulation is vital from this point of view with regards to the type of appliance prescription, type of teeth movement, and the mechanics of treatment.

In the present study, anterior teeth angulation and inclination were determined by the RaySet® device which is considered more accurate than the device used by Andrews [2]. It has been shown that there are no significant gender differences in many parameters, namely, anterior teeth angulation, inclination, Bolton ratio, overjet, and overbite, while highly significant gender differences were recorded in the linear measurements of the dental arch length and width being higher in males than females. This comes in agreement with the findings of many previous studies [19–21] as females possess smaller bony ridges and alveolar processes with a characteristic feature of late growth in males [18].

Pearson's correlation coefficient test showed that there was no significant correlation between angulation and inclination of anterior teeth with Bolton's ratios; this finding comes in agreement with the results of Alamir et al. [16] and Newaz et al. [22] who utilized the cephalometric radiograph to measure anterior teeth inclination and found a nonsignificant correlation between the inclination and Bolton's ratios. The present study depended on the anterior teeth crowns' inclination as taking radiographs for normal individuals had legal and ethical issues and the incisors' inclination from the radiograph differ from that measured on the models.

Angulation and inclination of upper central and lateral incisors did not correlate with arch width, while they significantly correlated directly with the total arch length, i.e., increased upper anterior teeth inclination will increase the dental arch length. This confirmed the result of Tuverson [11] who found that the lingual inclination of the root will add 1 mm to the arch length and O'Higgins et al. [15] who found that an increase in the maxillary incisors inclination by 5 degrees will increase the arch length by approximately 1 mm.

Hussel and Nanda [12] explained the role of maxillary incisors inclination considering the incisor as a rectangle viewed from the occlusal view, so “the arc formed by the incisal edge of maxillary teeth has a larger radius than the arc formed by the cervical part of the crown. If the incisal arch is not larger than the cervical one, there will be adverse arch length, so the arch length is influenced by the degree of inclination of maxillary incisors.” Moreover, Pontes et al. [13] confirmed the findings of the present study as an increase in anterior teeth angulation will produce a mild increase in the arch perimeter at the level of the crown (maximum 0.5 mm per incisor).

When O'Higgins et al. [15] studied the effect of maxillary incisors inclination on the dental arch length using natural and acrylic teeth with varying degree of inclinations, a direct relation was reported being greater with the natural incisors which may be related to the teeth size and morphology. They assumed that parallel-sided incisors showed the greatest increase in arch length, whereas the relatively triangular in shape incisors showed the smallest increase.

On the other hand, overjet and overbite were not correlated with the incisors' angulation and inclination in the present study, and this appears logical as the selected cases had normal occlusion with normal overjet, overbite, and perioral musculature, so mild skeletal discrepancy will be compensated by the upper and lower incisors to get normal overjet and overbite.

The major strength of the current study is taking individuals with normal occlusion, while Pontes et al. [13] and Jain et al. [14] utilized CBCT and working models, respectively. Teeth with normal occlusion have been compensated for the mild skeletal discrepancies and not hypothetical like in other studies. Further study with a large sample size is needed to evaluate the dental arch form and maxillary incisors size and morphology on their inclination in the dental arch and consequently on the length of the dental arch.

## 5. Conclusions

Within the limitations of the study like the small sample size, this study indicated that it is important to consider different factors such as angulation and inclination in addition to overbite, overjet, tooth size ratio, and arch dimensions in developing the diagnosis and treatment planning to get final optimal occlusion of the finished cases.

## Figures and Tables

**Table 1:** Descriptive statistics and gender differences for all measured parameters.

Parameters		Descriptive statistics				Gender difference	
		Males		Females			
		Mean	S.D.	Mean	S.D.	*t*-test	*P* value
Inclination (°)	U1	8.034	3.437	8.118	6.896	−0.053	0.958
	U2	6.638	3.095	7.183	6.168	−0.381	0.705
	U3	−2.785	4.684	−3.583	4.919	0.540	0.592

Angulation (°)	U1	3.300	2.162	2.785	2.921	0.666	0.509
	U2	6.083	1.783	5.456	2.161	1.041	0.304
	U3	4.748	2.182	3.873	2.536	1.213	0.232

Crown thickness (mm)	U1	2.167	0.189	2.151	0.234	0.242	0.810
	U2	2.214	0.198	2.168	0.233	0.702	0.487

Bolton's ratios	Anterior	78.627	2.448	78.334	3.138	0.344	0.733
	Overall	92.475	1.836	92.386	2.678	0.130	0.897

Arch width (mm)	Intercanine width	36.688	1.316	34.711	1.802	4.163	≤ 0.001
	Interpremolar width	49.668	1.712	46.628	2.130	5.187	≤ 0.001
	Inter-first molar width	54.708	2.004	51.322	2.717	4.708	≤ 0.001
	Inter-second molar width	60.852	2.575	58.011	3.455	3.092	0.004

Dental relation (mm)	Overjet	2.739	0.796	2.911	0.742	−0.720	0.475
	Overbite	2.304	0.942	2.717	0.675	−1.586	0.120

Arch length (mm)	Anterior arch length	10.712	0.977	10.550	0.885	0.557	0.580
	Molar vertical length	34.136	1.769	32.911	1.655	2.301	0.027
	Total arch length	46.732	2.465	44.794	2.004	2.743	0.009

**Table 2:** The correlation among different variables.

Parameters			Inclination			Angulation		
			U1	U2	U3	U1	U2	U3
Thickness	U1	*r*	0.174	0.193	0.031	0.111	0.058	0.019
		*P*	0.266	0.215	0.843	0.480	0.714	0.905
	U2	*r*	0.242	0.241	0.086	0.093	0.112	0.028
		*P*	0.119	0.120	0.582	0.555	0.476	0.858

Bolton's ratios	Anterior	*r*	0.084	0.185	0.119	−0.115	−0.009	−0.116
		*P*	0.592	0.236	0.447	0.461	0.954	0.458
	Overall	*r*	0.069	0.189	0.084	−0.208	−0.138	−0.151
		*P*	0.660	0.225	0.590	0.180	0.379	0.333

Arch width	Intercanine width	*r*	0.110	0.095	0.136	−0.143	−0.029	−0.052
		*P*	0.484	0.545	0.386	0.361	0.855	0.739
	Interpremolar width	*r*	0.050	0.011	0.033	0.066	0.131	0.202
		*P*	0.748	0.945	0.836	0.675	0.401	0.193
	Inter-first molar width	*r*	−0.055	−0.079	−0.058	0.155	0.173	0.177
		*P*	0.728	0.613	0.710	0.321	0.269	0.255
	Inter-second molar width	*r*	−0.087	−0.064	0.044	−0.075	−0.039	0.054
		*P*	0.579	0.683	0.779	0.632	0.803	0.730

Dental relation	Overjet	*r*	0.153	0.184	0.076	0.028	0.176	0.133
		*P*	0.327	0.237	0.628	0.857	0.260	0.394
	Overbite	*r*	−0.164	−0.186	−0.170	0.016	−0.003	−0.071
		*P*	0.292	0.232	0.276	0.920	0.983	0.652

Arch length	Anterior arch length	*r*	0.081	0.013	−0.080	0.081	0.014	−0.074
		*P*	0.605	0.933	0.608	0.605	0.929	0.639
	Molar vertical length	*r*	0.271	0.248	0.113	0.089	0.198	0.170
		*P*	0.079	0.109	0.472	0.569	0.204	0.276
	Total arch length	*r*	0.334	0.321	0.133	0.170	0.276	0.213
		*P*	0.029	0.036	0.394	0.277	0.074	0.170

U1, upper central incisor; U2, upper lateral incisor; U3, upper canine; *r,* Pearson's correlation coefficient; *p*, *p*robability value.

## Data Availability

The raw data used to support the findings of this study are available from the corresponding author upon request.

## References

[1] Cobourne M. T., DiBiase A. T. (2016). *Handbook of Orthodontics*.

[2] Andrews L. F. (1989). *Straight Wire: The Concept and Appliance*.

[3] Littlewood S. J., Mitchell L. (2019). *An Introduction to Orthodontics*.

[4] Andrews L. F. (1972). The six keys to normal occlusion. *American Journal of Orthodontics*.

[5] Roth R. H., Graber T. M., Swain B. F. (1985). Treatment mechanics for the straight wire appliance. *Orthodontics: Current Principles and Techniques*.

[6] Ricketts R. M., Bench R. W., Gugino C. F., Hilgers J. J., Schulhof R. J. (1980). *Bioprogressive Therapy*.

[7] Alexander R. G. W. (1986). *The Alexander Discipline: Contemporary Concepts and Philosophies*.

[8] McLaughlin R. P., Bennett J. C., Trevisi H. (2001). *Systemized Orthodontic Treatment Mechanics.*.

[9] Damon D. H., Graber T. M., Vanarsdall R. L., Vig K. W. (2005). Treatment of the face with biocompatible orthodontics. *Orthodontics: Current Principles and Techniques*.

[10] Severino F. N., Aldabalde A. N., Hidalgo M. V. (2020). *Pitts 21 “Our Method” Time and Space in Passive Self-Ligation Treatments*.

[11] Tuverson D. L. (1980). Anterior interocclusal relations Part I. *American Journal of Orthodontics*.

[12] Hussels W., Nanda R. S. (1987). Effect of maxillary incisor angulation and inclination on arch length. *American Journal of Orthodontics and Dentofacial Orthopedics*.

[13] Pontes L. F., Cecim R. L., Machado S. M., Normando D. (2015). Tooth angulation and dental arch perimeter-the effect of orthodontic bracket prescription. *The European Journal of Orthodontics*.

[14] Jain M., Vyas M., Singh J. R. (2017). Effect of crown angulation of maxillary incisor on the effective arch perimeter. *Journal of Clinical and Diagnostic Research*.

[15] O’Higgins E. A., Kirschen R. H., Lee R. T. (1999). The influence of maxillary incisor inclination on arch length. *British Journal of Orthodontics*.

[16] Alamir G., Tsay T. P., Manasse R. J. (2017). Reliability study of the Bolton analysis using dental models from cases passed by the American Board of Orthodontics Clinical Examination. *Journal of Dentistry & Oral Disorders*.

[17] Bolton W. A. (1958). Disharmony in tooth size and its relation to the analysis and treatment of malocclusion. *The Angle Orthodontist*.

[18] Al-Zubair N. M. (2015). Determinant factors of Yemeni maxillary arch dimensions. *The Saudi Dental Journal*.

[19] El Samad Younes S. A. (1984). Maxillary arch dimensions in Saudi and Egyptian population sample. *American Journal of Orthodontics*.

[20] Alvaran N., Roldan S. I., Buschang P. H. (2009). Maxillary and mandibular arch widths of Colombians. *American Journal of Orthodontics and Dentofacial Orthopedics*.

[21] Ahmed H. M. A., Ali F. A. (2012). Dental arches dimensions, forms, and the relation to facial types in a sample of Iraqi adults with skeletal and dental class I normal occlusion. *Journal of Baghdad College of Dentistry*.

[22] Nawaz R., Azeem M., Hashmi A. A., Mahmood H. S., Akram M. H., Moazzam M. (2018). Correlation between Bolton ratio and incisal inclination. *Pakistan Journal of Medical & Health Sciences*.

